# Exploring a New Theoretical Model to Explain the Behavior of Medication Adherence

**DOI:** 10.3390/pharmacy10020043

**Published:** 2022-04-01

**Authors:** Elizabeth Unni, Sun Bae

**Affiliations:** 1 Department of Social, Behavioral, and Administrative Sciences, Touro College of Pharmacy, 230 West 125th Street, Room 505, New York, NY 10027, USA; 2 Eshelman School of Pharmacy, University of North Carolina at Chapel Hill, 301 Pharmacy Lane, Chapel Hill, NC 27599, USA; sunwoo_bae@unc.edu

**Keywords:** medication adherence, theoretical model, hierarchical model, health literacy, illness beliefs, medication beliefs, self-efficacy

## Abstract

Medication adherence is essential for optimal therapeutic outcomes. However, non-adherence with long-term therapy is at 50%. Several theoretical models have identified several key factors that could explain medication adherence. Though numerous interventions have been developed based on these theoretical models, the success rates with interventions are not the best. This paper proposes a new Hierarchical Model for Medication Adherence. In this model, we propose medication adherence as a five-tier model with medication adherence as the desirable behavior on the top of the pyramid. From the bottom of the hierarchy upwards, the skills/beliefs/behaviors to be achieved are: health literacy, belief in illness (impacted by perceived susceptibility and severity of illness), belief in medicines (impacted by treatment satisfaction), and self-efficacy (impacted by social support). The model further proposes that each individual will achieve or already have these skills/beliefs/behaviors at various levels. Screening patients for these benchmarks will enable providers to decide where to target interventions.

## 1. Medication Non-Adherence

The World Health Organization [1] defines medication adherence as the extent to which a patient’s behavior of taking medications follows the agreed recommendations from the healthcare provider. When prescriptions are appropriate, non-adherence can cause serious consequences for both the patients and the healthcare systems [2]. Poor adherence to medicines is a global issue, especially for long-term therapy, with estimated non-adherence rates at 50% [1]. As per literature, 20–30% of prescriptions are never filled, and only 50% of prescribed medications are taken as recommended [3]. Studies have shown that the prevalence of the non-adherence problem varies by disease states and population [4,5,6,7,8,9,10,11,12]. For example, while non-adherence to medications for type II diabetes was 34.6%, it was 27.7% for hypertension, and 39.2% for seizure disorders [4]. At least 45% of type II diabetes patients showed uncontrolled A1C levels after they started medication therapy, and the biggest cause was medication non-adherence [5]. Cost-related non-adherence is especially high in chronic diseases like diabetes and hypertension [6]. The comorbidities and supplies associated with diabetes drive most of the cost of treatment [7]. Non-adherence rates of antihypertensive medications are 30% on average across the spectrum of insured patients [8]. For Medicaid patients, the level of medication adherence as measured by the proportion of days covered is 54% for diabetes, 58% for hypertension, 59% for congestive heart failure, 30% for dyslipidemia, 27% for asthma, and 45% for depression [9]. According to Jackevicious et al. [10], 24% of the patients discharged from hospital after treatment for heart failure or stroke do not fill their prescription within 7 days. Adherence to treatment for diseases such as HIV or cancer is affected by several factors, including complexity of regimen, relationship with the provider, social stigma, and social support [1,11]. The rate of adherence with cancer medications ranges from 16% for hematologic malignancies, to 100% for lymphoma [12]. 

Not only is medication non-adherence highly prevalent, it is also expensive. In the United States, medication non-adherence costs approximately $100 billion every year [3]. The annual revenue loss estimated by the global pharmaceutical sector is $564 billion, or 59% of the $956 billion of the total global pharmaceutical revenue [13]. As with the prevalence of non-adherence, the costs associated with non-adherence also vary with disease conditions. For example, in the US, the annual economic cost of non-adherence ranges from $3347 per patient to $19,472 per patient in cardiovascular disease, while, for diabetes mellitus, the range is from $1142 per patient to $7951 per patient [14]. One study showed the annual economic cost of cancer medication non-adherence at $119,416 per patient [14]. In 2010, the estimated annual cost of medication non-adherence in the UK in the five therapeutic areas of asthma, hypertension, diabetes, cardiovascular disease, and schizophrenia was £500 million [15].

The goal of this paper is to conceptualize a new theoretical model to explain the behavior of medication adherence. Since there are several systematic reviews on medication adherence, including the theoretical models that explain medication adherence and the interventions that were used to improve medication adherence, this paper will not be a review. Rather, it will highlight the findings from the literature to support the proposed model. The paper will outline the various theoretical models that are often used in medication adherence literature, followed by the interventions that are used and the lack of optimal interventions. This will be followed by the new proposed model and the rationale for the various constructs used in the model. Finally, the paper will discuss how the new model can be used by practitioners and researchers in developing interventions to improve medication adherence. 

## 2. Theoretical Models to Explain Medication Non-Adherence

With non-adherence being a global issue, there are numerous studies on medication adherence that aim to understand the causes of non-adherence. These studies have identified several theoretical models and factors that affect adherence to medications. Examples of these theories include the self-efficacy theory, the social cognitive theory, the health beliefs model, the theory of planned behavior, and the self-regulation theory. Some of the factors associated with adherence, as explained by these theories, include trust in the physician, self-efficacy, social support, attitude towards medications, belief in the medications, perceived behavioral control, intention to take medicines, subjective norms, perceived benefits and barriers to taking medicines, and self-regulation [9,16].

A review conducted by Holmes et al. [16] found that the self-efficacy model has been used as a predictor of medication adherence the most frequently, in 17 out of 19 studies. The self-efficacy model within the social cognitive theory framework has been used to explain medication non-adherence [17]. While self-efficacy is generally defined as confidence in one’s ability to change; within the context of medication adherence, it specifically refers to a belief in the patient’s ability to adhere to a prescribed medication regimen in “challenging” situations [18]. A classic example of a challenging situation where self-efficacy can be used is when an individual has been diagnosed with cancer [11].

Another widely-used model to explain medication adherence is the health belief model, which identifies perceived barriers, benefits, severity, and susceptibility as the factors to explain medication adherence. Beliefs in the healthcare system and medications are affected by counseling and information supplied by the healthcare providers about the medications [11]. Compared to the will to change in challenging situations, patients’ preconceived notions and beliefs about medicines and illnesses are more powerful factors in following treatment regimens for conditions such as diabetes or hypertension [11]. The Information-Motivation-Behavior (IMB) model is a subset of the social cognitive theory, and explains that providing patients with information that encourages health-promoting behavior facilitates motivation. Eventually this leads to more positive health behavior skills, like medication adherence [19].

The self-regulation theory, another often-used theory in adherence studies, is constructed with factors that affect treatment beliefs, such as medication harm and overuse in general, and the necessity and concerns associated with medications in a more specific manner [16]. According to this theory, patients who have expectations of positive outcomes from the treatment will better adhere to the medication [16]. Utilizing the Illness Perception Questionnaire (IPQ-R) and the Beliefs about Medicines Questionnaire (BMQ) is also useful in screening patients’ beliefs and feelings about medications and about their diseases. This helps detect the discrepancy between a patient’s belief and a doctor’s view of medicine [20]. 

Individuals with chronic disease conditions also need social support to adhere to their medicines. These supports can be functional, structural, and informational. Social support through daily interconnectedness and feedback mechanisms affects individual behavior, and these networks promote adherence to medications [21]. The social support in this model affects adherence through affective and cognitive factors [22]. 

Another crucial factor in medication adherence is health literacy, which is defined as the skills that can be used to improve or maintain health. The National Institute of Health [23] further defines health literacy as “the degree to which individuals have the capacity to obtain, process, and understand basic health information and services needed to make appropriate health decisions”. Paasche-Orlow and Wolf [24] have proposed that health literacy affects health outcomes in three ways: access to health care, interaction between patients and their health care professionals, and self-care. Chronic disease management requires assessment, comprehension, and utilization of health information; thus, health literacy will be helpful in explaining the health behavior of patients who have chronic conditions.

## 3. Interventions to Improve Medication Adherence

Many studies have proposed interventions to improve medication non-adherence, and the literature has systematic reviews examining these interventions [25,26,27]. However, the interventions for non-adherence in chronic conditions have not shown evidence for large-scale improvement [25,26,27,28,29,30]. A recent systematic overview of systematic reviews by Anderson et al. [27] reported low or very low evidence from 45 of the 50 systematic reviews. Similarly, a systematic review and meta-analysis by Conn and Ruppar [26] in 2017 reviewed 771 intervention studies and reported the modest effect size of 0.290. The authors recommended using more behavior-based intervention strategies, such as habit forming. Recently, researchers have tested many interventions, including non-restricted intervention types, special packaging, electronic reminders, dose simplifications, and cognitive behavior change techniques; it was concluded that these current interventions are not effective for chronic problems [27]. Most of the interventions used in the studies were technical and reward methods and were added practice on top of standard of care [28]. However, the majority of them were suggested to be short-term and ineffective when solely utilized. When educational, attitudinal, and technical interventions were used together as a multicomponent intervention, the effect lasted in the longer-term [28]. Another study indicated that multicomponent interventions were most effective in the longer-term, and included both attitudinal interventions (such as linking the adherence behavior to habits, giving feedback) and technical interventions (such as using special packaging, using pill boxes, self-monitoring of blood pressure) [29]. Educational intervention used in tandem with technical intervention had a positive effect on the improvement of medication adherence [30].

## 4. Lessons Learned from Behavioral Sciences

When studies have identified many factors that lead to non-adherence, but the interventions based on these factors are not as effective as expected, it is time to rethink adherence. Are we missing any crucial elements? Are we examining adherence as a behavior? From the literature, we know that adherence to medicines used for chronic conditions is a learned behavior. Once diagnosed with a chronic condition, an individual must commit to taking medicines as prescribed for the rest of their life. This is not a simple task, and is thus the reason for the high levels of medication non-adherence. So how does one develop a new behavior? Are there certain criteria that need to be satisfied before adherence behavior is learned? Is this the reason for the low success rates of interventions? Since we have identified many factors that affect medication adherence, are we targeting these factors haphazardly? Should there be a more formal structure for these interventions? 

When examining learning and actualization theories, foremost is Maslow’s hierarchy of needs model [31]. In this model, an individual attains self-actualization after achieving certain stages in life, such as physiological needs, safety needs, belongingness needs, and esteem needs. As such, we do not expect an individual to have needs of belongingness before their physiological and safety needs are met. Similarly, Clayton Alderfer’s existence-relatedness-growth (ERG) theory [32] states that for employee motivation, they have to go through three stages: existence (similar to Maslow’s physiological and safety needs), relatedness (similar to Maslow’s belongingness needs), and growth (similar to esteem and self-actualization). Can we identify such constructs in medication adherence literature? 

Bosworth et al. [33], in a recent publication, discuss the time-varying reasons for non-adherence using the ABC taxonomy of medication adherence. In the ABC taxonomy of medication adherence, a patient has to go through the phases of initiation, implementation, and discontinuation [34]. Bosworth et al. [33] comment that most intervention studies focus on the implementation phase, and thus miss initiation, the first essential phase in medication adherence. They also created the goal formation process using self-regulation to develop medication adherence behavior. Similarly, the Capability Opportunity Motivation–Behavior (COM-B) framework [35] postulates that medication adherence is influenced by capability, opportunity, and motivation. Again, the question arises: How do we develop these skills, and is there an order for their development?

## 5. New Theoretical Model Proposed

**Figure 1:**
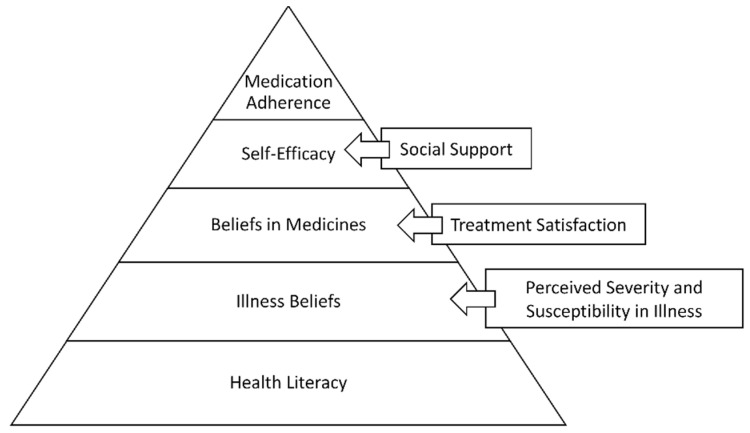
Hierarchical Model of Medication Adherence.

We propose Figure 1 as the Hierarchical Model for Medication Adherence (HMMA). Based on psychological theories such as Maslow’s hierarchy of needs, we would like to propose a model wherein an individual acquires certain skills/beliefs/behaviors at lower levels in order to achieve the higher level of medication adherence behavior. As in the initiation phase of the ABC taxonomy of medication adherence, patients need certain skills to initiate medication taking. What are the benchmarks all patients should have or achieve to be adherent with their medications? We also propose that each individual will achieve or already have these benchmarks at various levels. Screening patients for these benchmarks will enable providers to decide where to target interventions.

- According to this model, every individual should have adequate health literacy at the base level. This is the starting point for achieving the desired adherence behavior, as health literacy is needed for an individual to have a proper understanding of their disease and treatment. Without understanding their disease and medications, it can become very difficult for an individual with a chronic condition to maintain adherence behavior in the long run. The intervention review literature has also shown that patient education is one of the successful interventions.
- Once the patient understands their disease and treatment, the beliefs component comes into play. Does the patient believe in their illness? The common sense model of illness supports the hypothesis that a patient’s belief in their illness is a significant component of medication adherence. These illness beliefs can be further influenced by the variables in the health belief model, such as perceived susceptibility, perceived severity, demographics, and cues to action. If the individual has strong perceptions about their susceptibility to the disease and the severity of it, there is a high probability that the individual will be adherent to their medicines.
- The next phase in the model is an individual’s belief in their medicines. The beliefs in medicines framework argues that patients will weigh the necessity of taking medicines against the concerns they have about taking medicines. If the necessity outweighs their concerns, they will be adherent. This phase can be influenced by their treatment satisfaction, either real or perceived, for themselves or for someone else. If treatment satisfaction is high, beliefs in medicines may also favor the necessity side, and the patient will be adherent.
- The final stage of the hierarchical model is self-efficacy. Even if the individual has adequate health literacy and has positive beliefs, they still need to be self-efficient. They should be able to acquire and take their medicines as prescribed. This factor can be affected by the social support the patient has. For example, does the patient have a social support mechanism to remind them to take medicines as prescribed? Do they have a way to get to a pharmacy to pick up their medicines?

Thus, as seen in this proposed model, adherence interventions must be approached in a systematic way. As the reviews showed, self-efficacy is a strong predictor of medication adherence, likely because it is at the top of the pyramid, closer to adherence. However, interventions focused only on self-efficacy may not be efficient or effective if the patient does not have adequate health literacy or beliefs. Additionally, while one patient may struggle with health literacy, another may struggle with beliefs about medicines. It is therefore essential for health care providers to screen patients to understand which level they are on before starting interventions. The next section describes the literature to support the use of these variables in the Hierarchical Model of Medication Adherence (HMMA).

### 5.1. Health Literacy

Health literacy is the degree to which individuals have the capacity to obtain, process, and understand basic health information and services needed to make appropriate health decisions. This skill is essential for medication adherence. It is also among the cognitive strategies to improve medication adherence [26]. A meta-analysis that assessed the correlation between health literacy and medication adherence reported it at 0.14 [36]. The study also reported that interventions to improve health literacy increased adherence outcomes, with a correlation of 0.16. Zhang et al. [37], in their systematic review and meta-analysis, also report a small but statistically significant positive association between health literacy and medication adherence. They show that health literacy has a mediator relationship with other adherence determinants. Thus, we propose that health literacy is the initial process in this hierarchical model; though it is further away from medication adherence, it is still quite significant. Since patients need to understand their illness and the medicines to manage their illness, health literacy is critical. Additionally, educational interventions are one of the most effective interventions, and health literacy is needed for the success of these educational interventions [38]. It is also possible that key opinion leaders such as physicians or someone from the healthcare can impact health literacy, especially with new treatments and/or preventive treatments. 

### 5.2. Illness Perception

Illness perception is a term referring to the mental representations and personal ideas that people have about an illness [39]. Individuals shape their perceptions about their illness based on five components: beliefs about the identity of the illness, causes of illness, consequences of the illness, duration and cyclical nature of the illness, and controllability of the illness [40,41]. Studies have reported the significance of illness perceptions in important health outcomes, including medication adherence [39,40]. According to Elwy et al. [42], whether patients would seek treatment depended on their level of knowledge of the disease, their belief in the treatment’s efficacy, and their perceptions of the outcomes of the treatments. As such, we propose that for an individual to be adherent to medications, they need to acknowledge and understand their illness; thus, illness perception is the next step after health literacy in the hierarchical model. Since illness perceptions shape the overall decision-making process leading to medication adherence and health outcomes, the health belief model components, such as perceived susceptibility to the disease and perceived severity of the disease, play a significant part in influencing the illness perceptions [43].

### 5.3. Belief in Medicines

Once individuals believe that they have an illness that needs to be treated, the next step is their evaluation of the prescribed medications, which is influenced by necessity and concern beliefs about the prescribed medicines [2]. This framework is important since it affects the patient’s attitude and decisions about treatment [2]. The meta-analysis of 94 studies has shown a statistically significant relationship between necessity beliefs and adherence. As the standard deviation of a patient’s need for the treatment increases, the odds of adherence increase, whereas when the standard deviation of a patient’s concern for potential adverse outcomes increases, the odds of adherence decreases [2]. Thus, we propose belief in medicines as the next component of the hierarchy model. In other words, for an individual to take their medicine as prescribed, they need to believe in the medications. For this to happen, the treatment satisfaction with the current therapy or previous therapies can be a moderator.

### 5.4. Self-Efficacy

At this point in the model, the individual has the health literacy to understand their illness and medications. Once they develop the right perceptions about their illness and medicines, they need to have the skills to take their medicines as prescribed, and self-efficacy can play a significant role at this step. Self-efficacy is a component of social cognitive theory (SCT), heavily affected by personal factors that facilitate specific behavior to achieve the desired outcome [17]. Martos-Méndez [44] states that a patient’s self-efficacy has significant effects both directly and indirectly on medication adherence. With self-efficacy, where the patients feel confident in their ability accomplish certain outcomes, social support and satisfaction gained from social support also play an important role in increasing medication adherence [18]. In this way, self-efficacy influences patients at the visceral, cognitive, and motivational level [44]. Patient empowerment can positively impact their medication management. Thus, we propose that self-efficacy, moderated by social support, is the final step in this hierarchical model.

## 6. Application of the New Model

The proposed model is a way to rethink medication adherence as a behavior to be achieved. What are the steps need to achieve this behavior? From the past research, we know several factors that can impact medication adherence. How do we use it in the most efficient way to achieve the desired behavior? What is an algorithm that can be developed for the clinicians to check on the patient to understand their level of readiness to be adherent with the prescribed medicines?

Each component of the proposed hierarchical model closely correlates to medication adherence [2,36,37,39,40,45]. A recent publication by Russo et al., which used a Delphi process to identify the strongest psychological constructs from the literature, also included the proposed model constructs such as health literacy, illness perceptions, and treatment related beliefs as strong predictors. Self-efficacy was also identified, though not as strong as the other three variables [45].

However, from an intervention standpoint, we propose that each component is considered as a step to be achieved in order to move forward. For example, unless the individual achieves health literacy and believes in their illness and treatment, self-efficacy interventions cannot be optimal. Thus, similar to the psychological need models, we propose the Hierarchical Model of Medication Adherence, where to achieve medication adherence, several goals have to be met in a stepwise manner. The time and effort required to achieve each step in the model can vary for each individual. Some individuals may have already achieved certain steps. For example, there may be individuals who are already health literate and have accepted their illness, but need to achieve belief in medications to further progress. Another individual who trusts medicines may not believe they have the illness. For them, once they achieve the belief in illness step, they can move directly to the self-efficacy component. In other instances, there may be patients who are stuck at the self-efficacy part and need support. 

With this model, healthcare providers can ascertain the level for each patient using appropriate measurement instruments. Once the appropriate level is identified, interventions can be provided at that level. For example, a patient who already has accepted the illness does not have to be educated on the illness again, and efforts can be focused on educating them about the medicines. A patient who is struggling with self-efficacy can be provided interventions to improve self-efficacy, including social support. Schommer et al. [46] developed the Adherence Predictive Index (API), which focuses on patient centered communication, the preferred communication style which will motivate patients to be adherent. Healthcare providers can use the API at each intervention level to optimize the adherence behavior. Table 1 provides a list of the various screening tools and potential interventions that can be used for the HMMA model. This is not a comprehensive list, but rather a tool for the reader to start thinking. 

This model has not been empirically tested, though literature and previous studies by the authors have shown the potential for this model [45,47,48,49]. Further empirical studies need to be done to test the hypotheses proposed by this model. As complex as medication adherence is, there is the possibility that the model will be different for different therapeutic classes, especially between symptomatic and asymptomatic treatments. For example, in a symptomatic condition such as asthma or cancer, health literacy may be a stronger predictor than patient beliefs. However, for an asymptomatic condition such as cholesterol, patient beliefs may be the strong predictor compared to health literacy. As such, the variables in each phase in this hierarchical model should not be considered as causal relationships, but rather as stages that should be accomplished to reach the level of medication adherence.

## 7. Conclusions

Medication non-adherence is a complex phenomenon and learning behavior. Though several factors predict medication adherence, and several interventions have been implemented to improve medication adherence, this paper proposed a new theoretical model where medication adherence is considered a behavior that can be achieved by passing through various stages in a hierarchical fashion.

## Figures and Tables

**Table 1:** Examples of applying the HMMA model in practice and research.

Model Constructs	Screening Tool Examples	Potential Intervention Strategy
Health literacy	European Health literacy Questionnaire Test of Functional Health Literacy in Adults Rapid Estimate of Adult Literacy in Medicine Comprehensive Health Activities Scale Single Item Literacy Screener	Teach back method Visuals AHRQ Health Literacy Kit
Illness perceptions	Illness Perception Questionnaire	Education Cognitive Treatment Eliciting beliefs
Belief in medicines	Belief about Medicines Questionnaire Treatment Satisfaction Questionnaire	Education Motivational Interviewing Changes of Stage
Self-efficacy	Self-efficacy for Appropriate Medication Use Scale Long-term medication behavior self-efficacy Scale Medication Understanding and Use Self-Efficacy Scale	Education Social Support Telehealth Mobile applications
